# Factors associated with using alternative sources of primary care: a cross-sectional study

**DOI:** 10.1186/s12913-019-4743-4

**Published:** 2019-12-04

**Authors:** Charlie Reed, Felicia A. Rabito, Derek Werthmann, Shannon Smith, John C. Carlson

**Affiliations:** 1 Tulane School Public Health and Tropical Medicine, 1440 Canal St, New Orleans, LA 70112 USA; 2 Tulane School of Medicine, 1430 Tulane Ave, New Orleans, LA 70112 USA

**Keywords:** Access, Utilization, Racial/ethnic disparities, Distance, Perceived quality, Primary care, GIS

## Abstract

**Background:**

Mobile (MHCs), Community (CHCs), and School-based health clinics (SBHCs) are understudied alternative sources of health care delivery used to provide more accessible primary care to disenfranchised populations. However, providing access does not guarantee utilization. This study explored the utilization of these alternative sources of health care and assessed factors associated with residential segregation that may influence their utilization.

**Methods:**

A cross-sectional study design assessed the associations between travel distance, perceived quality of care, satisfaction-adjusted distance (SAD) and patient utilization of alternative health care clinics. Adults (*n* = 165), child caregivers (*n* = 124), and adult caregivers (*n* = 7) residing in New Orleans, Louisiana between 2014 and 2015 were conveniently sampled. Data were obtained via face-to face interviews using standardized questionnaires and geospatial data geocoded using GIS mapping tools. Multivariate regression models were used to predict alternative care utilization.

**Results:**

Overall 49.4% of respondents reported ever using a MCH, CHC, or SBHC. Travel distance was not significantly associated with using either MCH, CHC, or SBHC (OR = 0.91, 0.74–1.11 *p* > .05). Controlling for covariates, higher perceived quality of care (OR = 1.02, 1.01–1.04 *p* < .01) and lower SAD (OR = 0.81, 0.73–0.91 p < .01) were significantly associated with utilization.

**Conclusions:**

Provision of primary care via alternative health clinics may overcome some barriers to care but have yet to be fully integrated as regular sources of care. Perceived quality and mixed-methods measures are useful indicators of access to care. Future health delivery research is needed to understand the multiple mechanisms by which residential segregation influences health-seeking behavior.

## Background

Use of primary care services significantly reduces chronic disease morbidity. Adults in the U.S. who report seeing a primary care physician have lower odds of premature death and lower personal medical costs than those who report seeing specialists for their care [1]. Underutilization of primary care services is predominantly experienced in minority and low socioeconomic populations, two groups subject to high rates of chronic conditions [2, 3]. This is likely because these groups experience more barriers to accessing health services than other populations [4, 5]. Identifying and removing these barriers to access is critical to improving the health status of racially-marginalized and socially disadvantaged individuals.

Preliminary research suggests that residential segregation is a prominent barrier to accessing primary care [6–10]. Across the U.S., the odds of having a primary care physician shortage were 67% higher in majority African American zip codes than in zip codes with a White majority and the difference increased with increasing degree of segregation [8]. Geographic inaccessibility, primarily defined as distance, has been highlighted as a key mechanism by which residential segregation limits access [6, 8–10]. Establishing primary care clinics in minority neighborhoods has been shown to improve geographic access and subsequently increase utilization [10].

Among individuals living in segregated neighborhoods, the impact of geographic distance on access may be complicated by an individual’s perception of the quality of care available to them. As a result of segregation, minority individuals experience constrained social networks and a proliferation of medical mistrust, perceived discrimination, and perceptions of poor clinic quality [8]. These are additional barriers that make people less likely to seek and use primary care services that would otherwise be considered accessible [7, 11–13]. Additionally, people are known to travel farther in order to receive care from a doctor of their own race [14, 15]. Given this information, it appears that travel distance is important but subjective and that additional factors should be considered in order to more accurately identify the drivers of underutilization.

Community (CHCs), mobile (MHCs), and school-based health clinics (SBHCs) are community-based alternatives to traditional primary care. Their goal is to provide more geographically accessible care in trusted and less stigmatized settings [16–20]. Persistent barriers to traditional primary care sources suggest that alternative health care settings may have an important role to play in reducing health disparities by providing more accessible care. These alternative sources of health care are not typically studied as a health system. The goal of this study was to explore the extent to which MHCs, CHCs, and SBHCs are utilized by marginalized populations and to assess the factors associated with geographic residential segregation that influence their utilization.

## Methods

### Study setting and study population

The study was conducted in New Orleans, Louisiana where a long history of residential segregation continues to this day [21, 22]. Based on 2010 U.S. Census data, New Orleans ranks 32nd among Metropolitan areas for degree of residential segregation. Following the devastation of Hurricane Katrina in 2005, the New Orleans primary care system was decentralized with an emphasis on addressing racial and income differences in geographic access to care [23, 24]. A prominent feature of the reorganized health system was the integration of CHCs (including Federally-Qualified Health Centers), MHCs, and SBHCs in underserved communities. The new system reached an estimated 80% of the low-income population of the Greater New Orleans (GNO) area in 2012 [24, 25].

To obtain information about the extent to which CHCs, MHCs, and SBHCs are utilized, the factors that are associated with their use, and how these alternative forms of health care delivery are perceived by the community they are designed to serve, a convenience sample of 299 individuals was surveyed between August 2014 and December 2015. Eligible participants included adults (age ≥ 18), caregivers of children aged 5–17, and caregivers of adults who resided in New Orleans and who spoke Spanish or English.

### Data collection

Data were collected at community health fairs held throughout low-income and minority neighborhoods in the GNO area and during home visits as part of an ongoing asthma study targeting the same population. New Orleans’ health fairs are tabled by many organizations engaged in improving quality of life within the community. During the fairs, a table was staffed by trained researchers to provide and assist respondents the survey questionnaire. Participants were also recruited from an on-going asthma study. For these individuals the survey questionnaire was completed in their home. The 57-item questionnaire (Additional file 1), included socio-demographic characteristics, perceptions of CHC, MHCs, SBHCs, barriers to care, usual source of health care, presence of chronic diseases, connectivity of care, and health care utilization. All responses were self-reported. The survey instrument was pilot tested prior to its use and informed consent was obtained prior to any data collection. The study was approved by the Tulane University Biomedical Institutional Review Board. A detailed description of survey components follows.

### Geographic access

Geographic access was measured by mapping the shortest network distance (miles) via Orleans, Jefferson, and St. Bernard Parish (County) roads between the participant’s reported home address and the nearest eligible clinic (CHCs, MHCs, or SBHCs). Data were geocoded using ArcMap software version 10.2 (ESRI, 2011 Redlands CA). Home addresses outside of any of the 3 included parishes were excluded from the study. If an individual provided a street name but no house number, the midpoint of the street was used as the home address. A list of primary clinics active in 2014 was obtained from the Louisiana Public Health Institute. Clinics included 31 CHCs, 14 pediatric clinics (PCHCs), 4 MHCs, and 6 SBHCs. Adult and caregivers of adults were considered eligible to receive service from CHCs and MHCs, while children were considered eligible for PCHCs, MHCs, and SBHCs. Neither multiple buffer zones nor Euclidean distances were used to determine geographic access because network travel distance is considered a more precise representation [26–28].

### Perceptions of clinic quality and barriers to care

Study participant’s perceptions of alternative types of primary care clinics (CHCs, MHCs, SBHCs) was assessed using 20 questions from the Barriers to Care Questionnaire (BCQ). The reliability and validity of the BCQ has been established in previous studies [29]. The BCQ quantifies the “circumstances that may interfere with accessing or using care in low-income populations” [30, 31]. The BCQ is comprised of four subscales. The Pragmatics subscale measures logistical and cost issues that might prevent or delay appropriate utilization of a clinic. The Skills subscale measures the respondent’s perceptions of the acquired or learned strategies that are needed to navigate through, manipulate, or function competently within the health care system. The Expectations subscale measures the expectation that, if used, an individual will receive poor quality of care, and the Marginalization subscale measures the internalization and personalization of negative experiences within the health care system [30]. Participants answered questions for each clinic type, regardless of whether or not they had ever received services from that type of clinic. The tool was developed to score responses on a scale from 0 (item perceived to be a big problem) to 4 (not a problem at all) and then multiplied by 25 to generate a more communicable 0–100 scale; higher scores indicate fewer perceived barriers to care and higher perceived quality [29]. The survey generates a total score between 0 and 100 which is the calculated average across the average scores of the four subscales.

### Perceptions of quality of care and distance: mixed-methods measure

The absolute distance to a health clinic may be an inadequate predictor of clinic utilization [14, 32, 33]. The Satisfaction-Adjusted Distance (SAD) index was developed by Kwan & Hawthorne as a mixed-methods measure and alternative indicator [34]. The SAD index accounts for perceived quality of care and geographic distance simultaneously by adjusting travel distance by an individual’s perception of the clinic [34]. Perceived distance measures like SAD are novel but have been found to be useful in mapping multi-factorial concepts of the built environment [33, 35–37]. In the current study, BCQ score was substituted for the patient-satisfaction questionnaire score used in the development of SAD [34]. Both questionnaires measure similar domains (trust, interaction, skills, accessibility) using Likert scales with near identical score ranges (0–100 and 1–100 respectively). A lower than average BCQ score adds to the mapped travel distance, using the equation below.

$$ \mathrm{SAD}=\left[0.1\ast \left(\mathrm{Mean}\ \mathrm{BCQ}-\mathrm{Individual}\ \mathrm{BCQ}\right)\right]+\mathrm{Travel}\ \mathrm{Distance} $$

Separate equations were used to calculate adult and child scores. The SAD is presented as a continuous variable in all analyses. A higher SAD indicates less perceived access to care due to a combination of the perception of poor clinic quality and longer travel distances.

### Clinic utilization

The outcome variable in all models was clinic utilization. Adults and caregivers of adult dependents were considered utilizers if they answered ‘yes’ to having ever used a MCH or a CHC. Child dependent caregivers were considered utilizers if they answered ‘yes’ to having ever used a CHC, a MCH, or a SBHC. Non-users for both groups answered ‘no’ to using any of the eligible clinics.

#### Statistical analysis

Means and standard deviations are presented for continuous variables and the number and proportions are presented for categorical variables. The outcome variable, clinic utilization, was dichotomized in all analyses. Bivariate logistic regressions were performed to examine the independent relationship between clinic utilization and three indicators of perceived access (geographic distance, BCQ score, and SAD). Covariates, including the respondent (adult/adult responding for a child), gender, presence of a chronic condition (yes/no), insurance status, and measures of continuity of care; length of time visiting a place for care (No place, 0–3, 4–7, 8 or more years), and the length of time visiting a person for care (No person, 0–3, 4–7, 8 or more years), were considered as possible confounding variables. Any variable found to be significantly associated (*p* <  0.05) with clinic utilization in bivariate analyses was included in multivariate models to control for potential confounding. The primary exposure variables, BCQ score, travel distance, and SAD index were modeled separately. Unadjusted and adjusted odds ratios (OR) with 95% confidence intervals (CI) are presented to identify significant predictors of utilization. Correlation and linear regression analyses were performed between SAD index and its two components; BCQ total score, and travel distance. Median BCQ subscales, stratified by clinic type and whether the respondent had ever used the clinic, are presented to explore barriers by specific clinic type. All analyses were performed with SAS statistical software version 9.4 (SAS Institute, Inc. Cary, North Carolina); two-sided tests were assumed and *p*-values < 0.05 were considered statistically significant.

## Results

A total of 299 individuals completed the survey. Due to the low number of respondents (*n* = 7), caregivers of adult dependents were excluded from the analysis resulting in a sample size of 292. Most individuals were sampled from seven locations in Orleans Parish that hosted reoccurring health fairs. Two hundred and seventy-five survey respondents provided their home address and 256 (93.1%) were successfully geocoded. Of 55 clinics identified, all but two (96%) were successfully geocoded. Nineteen home addresses were not geocoded, including 15 not located within the Greater New Orleans area, three P. O boxes, and one car.

Characteristics of the study population, stratified by type of respondent, are presented in Table 1. The majority of respondents were African American (84.4%), female (67.0%) and between the ages of 40 and 64 years. Approximately 57% reported health care access information for themselves while the remaining responded on behalf of the child in their care. The high proportion of people with insurance was a surprise. The majority of adults (53.9%) reported having private health insurance while most children (80.2%) reported coverage through Medicaid.

**Table 1:** Population Characteristics

Characteristics (*N* = 292)	Type of Respondent n (column %)		
	Adult	Adult for Child	
Race/Ethnicity *n* = 289			
African American	131 (79.4)	113 (91.1)	
Other	34 (20.6)	11 (8.9)	
Gender *n* = 285			
Female	131 (79.9)	60 (49.6)	
Age in years *n* = 281			
< 18	–	125 (100.0)	
18–39	46 (29.5)	–	
40–64	88 (56.4)	–	
≥ 65	22 (14.1)	–	
Type of Insurance *n* = 258			
Medicaid	19 (13.4)	93 (80.2)	
Medicare	20 (14.1)	3 (2.6)	
Private	91 (64.1)	20 (17.2)	
Uninsured	12 (8.5)	0 (0.0)	
Years living at current address *n* = 270			
0–2	35 (23.2)	53 (44.5)	
2–4	17 (11.3)	34 (28.6)	
4–12	36 (23.8)	29 (24.4)	
> 12	63 (41.7)	3 (2.5)	
Time visiting a specific health facility, *n* = 267			
No regular Doctor/Nurse	8 (5.4)	2 (1.7)	
0–3 Years	63 (42.6)	42 (35.3)	
4–7 Years	52 (35.1)	69 (58.0)	
> 8 years	25 (16.9)	6 (5.0)	
Time visiting a specific health care provider, n = 267			
No regular Doctor/Nurse	21 (14.0)	4 (3.4)	
0–3 Years	67 (44.7)	38 (32.2)	
4–7 Years	39 (26.0)	69 (58.5)	
> 8 years	23 (15.3)	7 (5.9)	
Chronic condition *n* = 255			
No Chronic Condition	39 (26.7)	24 (22.0)	
Any Chronic Condition	107 (73.3)	85 (78.0)	
Perceive access for chronic conditions *n* = 194			
Access	91 (84.3)	79 (91.9)	
No/Incomplete Access	17 (15.7)	7 (8.1)	
Perceive General Access *n* = 280			
Access	138 (85.7)	115 (96.6)	
No Access	23 (14.3)	4 (3.4)	
Usual Source of Care *n* = 278			
Private	82 (51.9)	103 (85.8)	
Other	58 (36.7)	16 (13.3)	
None/ER	18 (11.4)	1 (0.9)	
Utilization of any 3 clinic subtypes n = 267			
Used a clinic	93 (62.0)	39 (33.3)	
Have not used a clinic	57 (38.0)	78 (66.7)	
Continuous Covariates	Statistic		
	Median	SD	Range
Time at current address (years)	4.00	13.80	80.04
Travel Distance (miles)	1.30	1.28	5.67
BCQ Total Score	64.76	22.72	87.29
Satisfaction-adj. Distance	1.94	2.56	12.75

Respondents had a stable residential history reporting, on average, living 10.4 years at their current address. Continuity of health care was also unexpectantly high; 87.3% of respondents reported receiving health care at the same location for between 1 and 7 years. Notably, continuity of care was higher for the place where health care was received than for the specific provider of their care. Nine percent of individuals lacked a specific provider for care, compared to 3.7% lacking a place for care. The proportion of respondents reporting no regular health care provider was more than double the proportion reporting no regular place of care.

Perceived access to care for general health needs was high (90.4%). Chronic conditions were common in both children and adults with approximately one-third of all adults and children reporting currently having a chronic condition. Allergies was the most prevalent condition among children (*n* = 49) and hypertension or high blood pressure was the most prevalent condition among adults (*n* = 22) (data not presented). Overall, of those with a chronic condition, 87.7% reported having access to health care for their specific condition. Perceived access to care for children with a chronic condition was high with 97% reporting having access to specialized care. Overall, private clinics were the most common place for respondents to receive care and community clinics were the second most common source of care. The type of clinic used differed between adults and children; 85.8% of children received care in private clinics while 51.9% of adults received care from private clinics. Adults answering for themselves averaged higher perceptions of quality (69.1) than adults answering for children (62.9).

Overall, use of either CHCs, MHCs, or SBHS was high; 49.4% of all respondents reported using either a CHC, MHC or SBHC. The proportion of adults who reported ever using one of the three types of clinics was considerably higher (62.0%) than the proportion of children (33.3%). Community clinics were the most frequently used source (76.3%) compared to 40.9% using a MHC and 39.5% using a SBHC.

The average travel distance from home to the nearest clinic was 1.72 miles. The farthest travel distance to either a CHC, MCH, or SBHC was 5.76 miles. School-based clinics (median 56.67) had the lowest perceived quality followed by MHC (61.04) and CHCs (64.79). Perceived quality was significantly higher among those who had used the clinic compared to those who had not (data not shown). As shown in Table 2, pragmatic issues presented the greatest barrier to receiving quality care followed by feelings of marginalization. The greatest pragmatic issues (median < 75), consistent across clinic type, were “receiving care after hours or weekends” and “time spent waiting to be seen.” For MHC and SBHCs “having to take off work” was a prominent barrier. “Getting to care” did not score as a noteworthy pragmatic barrier for any clinic (median > 75). Among the marginalization items, “not knowing what to expect from one visit to the next” and “doctors rushing patients through the visit” were ubiquitous barriers. “Feeling that doctors provided as little service as possible” was also a barrier for MCH and SBHCs.

**Table 2:** BCQ score by Subscale and Clinic Type

BCQ Scale* (*n* = 280)	Clinic Type		
	Mobile	Community	School-based
Pragmatics	58.33	58.33	54.17
Skills	66.67	66.67	58.33
Marginalization	55.00	63.75	55.00
Expectations	66.67	75.00	58.33
Total**	61.04	64.79	56.67

* BCQ scored on scale 0–100, higher scores indicate greater perceived quality

** Total Score is calculated as an average across all subscales

When travel distance was adjusted for patient satisfaction using the SAD index, the average travel distance to a clinic was perceived to be 1.79 miles and the farthest perceived distance was 9.68. Compared to caregivers answering for children, adults had a shorter average travel distance to the nearest clinic (1.4 vs. 2.0 miles) and lower average SAD score (1.2 vs. 2.4).

Table 3 presents the bivariate association between predictors of access to health care and utilization. Higher perceived quality, measured by total BCQ score, was significantly associated (OR 1.02; 95% CI 1.01–1.04) with increased utilization. Higher SAD score (indicating less perceived access) was negatively associated (OR 0.81; 95% CI 0.73–0.91) with utilization. Travel distance, measured in miles, was not independently associated with clinic utilization (OR = 0.91, 95% CI 0.74–1.11). The type of respondent (adult vs. child caregiver), insurance status, the length of time having visited one place, and having the same provider were all significantly associated with clinic utilization.

**Table 3:** Bivariate Analyses of health care utilization

Covariates *N* = 224	Utilization			
	Used n (col. %)	Not used n (col. %)	OR (95% CI)	*p*-value
Type of Respondent				
Adult	77 (67.0)	40 (37.7)	3.50 (2.02–6.06)	< 0.01
Child Dependent	38 (33.0)	69 (63.3)		
Gender				
Male	33 (29.2)	44 (41.1)	0.59 (0.34–1.03)	0.06
Female	80 (70.8)	63 (58.9)		
Presence of a Chronic Condition				
Any Chronic Condition	79 (78.2)	79 (79.8)	0.91 (0.46–1.80)	0.78
No Chronic Condition	22 (21.8)	20 (20.2)		
Insurance Status				
Medicaid	38 (37.3)	60 (58.8)	0.51 (0.28–0.94)	< 0.01
Medicare	15 (14.7)	6 (5.9)	2.02 (0.71–5.78)	
Uninsured	7 (6.9)	2 (2.0)	2.83 (0.55–14.54)	
Private	42 (41.2)	34 (33.3)	Reference	
Length of Time Visiting a Specific Place for care*				
No Regular Doctor/Nurse	5 (4.7)	2 (1.9)	0.94 (0.14–6.20)	< 0.01
0–3 years	51 (47.6)	35 (33.3)	0.55 (0.20–1.53)	
4–7 Years	35 (32.7)	62 (59.1)	0.21 (0.08–0.59)	
> 8 Years	16 (15.0)	6 (5.7)	Reference	
Length of Time Visiting a Specific Person for care*				
No Regular Doctor/Nurse	13 (12.04)	7 (6.7)	0.62 (0.16–2.43)	< 0.01
0–3 years	49 (45.4)	34 (32.4)	0.48 (0.16–1.45)	
4–7 Years	31 (28.7)	59 (56.2)	0.18 (0.06–0.53)	
> 8 Years	15 (13.9)	5 (4.8)	Reference	
Measures of access	Used Clinic Median (SD)	Not used Median (SD)	OR (95% CI)	p-value
Travel Distance (miles)	1.31 (1.19)	1.21 (1.36)	0.91 (0.74–1.11)	0.35
BCQ Total Score (0–100)	73.4 (23.1)	55.4 (19.3)	1.02 (1.01–1.04)	< 0.01
Satisfaction-adjusted distance	1.22 (2.56)	2.49 (2.36)	0.81 (0.73–0.91)	< 0.01

* BCQ scored on scale 0–100, higher scores indicate greater perceived quality

** Total Score is calculated as an average across all subscales

The results of multivariable regression models are presented in Table 4. Both perceived quality (aOR = 1.02, 95% CI 1.01–1.04) and SAD (aOR = 0.84, 95% CI 0.74–0.96) remained significant predictors of utilization in models adjusted for insurance status, adult or caregiver respondents, length of visit place and person. Both BCQ and travel distance accounted for a significant proportion of the variance in SAD, 74.7 and 24.9% respectively (data not presented).

**Table 4:** Multivariable models of health care utilization

Covariates	aOR (95% CI) *p*-value**			
	Model 1* (BCQ)		Model 2* (SAD)	
Type of Respondent (vs Child Caregiver)				
Adult	2.21 (0.92–5.28)	0.08	1.95 (0.814–4.68)	0.13
Insurance (vs. Private)				
Medicaid	0.88 ((0.36–2.13)	0.63	0.79 (0.33–1.91)	0.51
Medicare	1.28 (0.40–4.11)	0.72	1.23 (0.38–3.97)	0.71
Uninsured	1.19 (0.16–8.82)	0.90	1.17 (0.16–8.81)	0.87
Length of Visit Place (vs. > 8 years)				
No Regular	1.21 (0.06–26.02)	0.61	0.91 (0.04–18.97)	0.78
0–3 years	0.77 (0.08–7.31)	0.99	0.75 (0.08–6.89)	0.90
4–7 years	0.39 (0.04–3.97)	0.19	0.38 (0.04–3.80)	0.23
Length of Visit Person (vs. > 8 years)				
No Regular	0.57 (0.04–7.99)	0.55	0.53 (0.04–7.32)	0.56
0–3 years	0.98 (0.09–11.02)	0.60	0.83 (0.08–9.09)	0.74
4–7 years	0.69 (0.05–8.74)	0.80	0.63 (0.05–7.89)	0.78
Quality of Care Measures				
BCQ	1.02 (1.01–1.04)	< 0.01	–	–
SAD	–	–	0.85 (0.74–0.97)	< 0.01

* Model 1 includes BCQ as the primary exposure variable; Model 2included SAD as the primary exposure variable

**Adjusted for type of respondent, insurance status, and length of time visiting a specific place for care or provider of care

- Variable was not included in the model

## Discussion

This study sought to explore the extent to which MHCs, CHCs, and SBHCs are utilized by the populations for which they were designed to serve and to assess the factors associated with geographic residential segregation that influence their utilization. Approximately half of the surveyed respondents had used a community, mobile, or school-based clinic, personally or as an accompanying caregiver. Perceptions of clinic quality and satisfaction-adjusted distance were statistically significant predictors of clinic utilization, while geographic distance to a clinic was not. The high frequency of care utilization in our study was consistent with other studies that indicate an overall improvement of the New Orleans health care landscape since Hurricane Katrina [24, 38]. However, most survey respondents sought regular care at a private clinic. There were surprisingly high proportions of individuals with private insurance coverage and good continuity of care amongst our sample. Collectively, this evidence suggests that use of CHCs, MHCs, and SBHCs may be more intermittent and unexpected in nature, filling gaps in care that traditional sources cannot. More studies are needed to explore these findings.

Travel distance, though not a significant predictor of utilization, was still an important component of access. Research shows that in some cases distance fails to conceptualize the burden of travel that should be included in the definition of geographic access. Limited access to a motor vehicle, chronic illness, and familial responsibilities all increase the burden of travel and are negatively associated with health care utilization and access [39, 40]. Racial disparities in travel burdens are also well-documented [41]. For example, 20 % of New Orleans households do not own a car, and these households are concentrated in low-income and minority neighborhoods [42, 43]. Because distance does not capture these sentiments, it alone is not a complete measure of accessibility among marginalized populations.

Individuals who perceived an alternative clinics to be of higher quality were significantly more likely to have used a clinic than those who perceived them to be of lower quality. Feelings of marginalization was usually the lowest scoring subscale of the BCQ (second lowest for CHCs). It is evident that distance misrepresents racialized spaces as fixed at home. Social networks constrained by residential segregation rarely include individuals with positive health insight, and likely perpetuate negative experiences or medical mistrust [44, 45]. In New Orleans word of mouth was by far the preferred method to receive information about health care [46]. Pragmatics was another low scoring subscale of the BCQ; survey respondents commonly felt rushed or ignored and that getting seen by a health professional is too time consuming. The benefits of using a clinic close to you may be overshadowed by the quality and timeliness of care delivery. These perceptions of alternative clinics as marginalizing and pragmatically challenging are interesting and they contradict the established literature. It appears that the alternative clinics have yet to fully integrate into the communities they serve.

The mixed-methods predictor, SAD, combines distance and perceptions of quality into one significant predictor of health care utilization. Individuals with a greater perceived distance were significantly less likely to have ever used a clinic than those with a lesser perceived distance. As a measure of access, SAD captures the social and physical barriers to care that stem from residential segregation. More researchers are calling for mixed-method measures that revisit the geographic roles of space and place when studying segregation, health, and accessibility [35, 47].

Strengths of the study include a descriptive and analytic analysis of an alternative health system, a patient-centered characterization of marginalized populations, and a comparison of multiple measures of access to care. The study has several limitations. Although we identified various factors associated with utilization, the cross-sectional design prevents assessing whether they predict utilization. The use of a convenience sample and the restriction of the sample to New Orleans may limit generalizability of the findings. The high utilization rates, continuity of care, and private insurance coverage may be an artifact of the sampling method in that those attending health fairs may over represent individuals currently engaged in the health care system. Measuring distance to the nearest facility based on home address is another limitation as individuals may use clinics that are more proximal to other locations in their life.

## Conclusion

Community, Mobile, and School-based clinics may be equipped to overcome physical and social barriers to care but have yet to fully integrate into neighborhoods as regular sources of care. Distance is not sufficient to predict the use of a primary care clinic. An individual’s perceptions of clinic quality significantly predicted clinic usage, and when combined with distance is a useful indicator of utilization of health care services. Future research regarding access to health services in residentially segregated populations needs to consider the many mechanisms by which segregation can influence health seeking behavior.

## Supplementary information

**Additional file 1.** Questionnaire. This is a copy of the 57-item questionnaire referenced in the manuscript. It includes the previously cited Barriers to Care Questionnaire (starting at Question #17) that was developed and validated by Seid et al. [29]. The items prior to Question 17 were designed by the authors of this manuscript.

## Data Availability

The datasets generated and analyzed during the current study are not publicly available due to identifying information (addresses) and HIPAA requirements.

## Abbreviations

aOR: Adjusted Odds Ratio
BCQ: Barriers to Care Questionnaire
CHC: Community Health Clinic
CI: Confidence Interval
GNO) area: Greater New Orleans
MHC: Mobile Health Clinic
OR: Odds Ratio
SAD: Satisfaction-adjusted Distance
SBHC: School-based Health Clinic
