# Supplementary material for: Factors associated with using alternative sources of primary care: a cross-sectional study
Source: BMC Health Serv Res. 2019 Dec 4;19:933. doi: 10.1186/s12913-019-4743-4 (PMC6894211; doi:10.1186/s12913-019-4743-4)
Supplement: Supplementary file 1 — Additional file 1. Questionnaire. This is a copy of the 57-item questionnaire referenced in the manuscript. It includes the previously cited Barriers to Care Questionnaire (starting at Question #17) that was developed and validated by Seid et al. [29]. The items prior to Question 17 were designed by the authors of this manuscript. [file 12913_2019_4743_MOESM1_ESM.pdf]

Access to Care Questionnaire

1. What is your age range? [CIRCLE ONE]

18-39		40-64		65+	

2. What is your gender?

1☐ Male

2☐ Female

3. What is the highest grade or school level that you have completed?

1☐ Less than a high school degree

2☐ High school degree

3☐ More than a high school degree

4. Are you Hispanic /Latino?

1☐ No, not Hispanic/Latino

2☐ Yes, Hispanic/Latino

5. What is your race? [SELECT ONE OR MORE OF THESE CATEGORIES]

1☐ White

2☐ Black or African American

3☐ Asian

8☐ Other **Specify:** a. ______________________

6. What is your current home address?

Address: ____

City, State, Zip: __________________________________

7. How long have you lived here? ___________________________

8. What insurance do you have? [CHECK ALL THAT APPLY]

1☐	Medicaid	→ Does he/she have a primary care provider?	1☐ Yes	2☐ No
2☐	Medicare	→ Does he/she have a primary care provider?	1☐ Yes	2☐ No
3☐	Private	→ Does he/she have a primary care provider?	1☐ Yes	2☐ No
4☐	Uninsured	→ Does he/she have a primary care provider?	1☐ Yes	2☐ No
0☐	Other, please specify: a. ________________________________			
		→ Does he/she have a primary care provider?	1☐ Yes	2☐ No

9. Do you have any of these health conditions? [CHECK ALL THAT APPLY]

99☐	None → skip to #12	8☐	Hypertension/High Blood Pressure
1☐	Diabetes Type I	9☐	Heart Disease
2☐	Diabetes Type II	10☐	Cancer
3☐	Asthma	11☐	COPD (Chronic Obstructive Pulmonary Disease)
4☐	Allergies	12☐	Arthritis
5☐	ADD/ADHD	13☐	Obesity
6☐	Kidney Disease	14☐	Anxiety/Depression
7☐	Seizure Disorder	0☐	Other ______________________

10. Do you feel you have access to care for these conditions (from question 9)?

1☐	Yes to all
2☐	Yes to some → a. comment: ____
3☐	No → skip to #12
8☐	Not Applicable

11. For these conditions, do you see a specialist or your primary care doctor?

1☐	Specialist , list conditions: ________________________________
2☐	Primary care doctor (PCP) , list conditions: ________________________
3☐	Both specialist and primary care doctor , list conditions: ____________________
4☐	I do not see a specialist or primary care doctor, list conditions: _________________
8☐	Not Applicable

12. Now, think about your overall health care needs. Do you feel you currently have access to general primary healthcare?

1☐	Yes
2☐	No

13. What type of clinic do you go to for your primary care?

1☐	Private clinic
2☐	Community clinic (for example: Daughters of Charity, St. Thomas, EXCELth, Jefferson Community Clinics, St. Charles Community Health Center)
3☐	Mobile clinic
8☐	Other _______________________
0☐	Don't know
9☐	None → skip to #16

14. If there is one particular place that you go for almost all of your health care, how long have you gone there?

1☐	Less than 1 year
2☐	1 – 3 years
3☐	4 -7 years
4☐	8 – 10 years
5☐	11 or more years
0☐	Don't Know
6☐	I don't have a regular doctor or nurse

15. If there is one particular person that you think of as your regular doctor or nurse, how long has this person been your doctor or nurse?

1☐ Less than 1 year

2☐ 1 – 3 years

3☐ 4 -7 years

4☐ 8 – 10 years

5☐ 11 or more years

0☐ Don't Know

6☐ I don't have a regular doctor or nurse

16. Have you ever used a mobile health clinic?

1☐ Yes → What Kind? a. ________________________________

2☐ No

Please answer #17, regardless of your answer to #16.

17. People often face barriers when trying to get health care. Do you think these would be problems going to a MOBILE HEALTH CLINIC?

**Problems with:**	Big problem	Somewhat of a problem	Unsure	Not a big problem	Not a problem at all
a. Getting to the mobile clinic	(0)	(1)	(2)	(3)	(4)
b. Contacting the mobile clinic by phone	(0)	(1)	(2)	(3)	(4)
c. Having to wait too many days to be seen	(0)	(1)	(2)	(3)	(4)
d. Getting care after hours or on the weekends	(0)	(1)	(2)	(3)	(4)
e. Having to take care of household responsibilities	(0)	(1)	(2)	(3)	(4)
f. Having to take time off work	(0)	(1)	(2)	(3)	(4)
g. Doctors or nurses not fluent in your language	(0)	(1)	(2)	(3)	(4)
h. Getting referrals to specialists	(0)	(1)	(2)	(3)	(4)
i. Getting enough help with paperwork or forms	(0)	(1)	(2)	(3)	(4)
j. Mistakes made by doctors or nurses	(0)	(1)	(2)	(3)	(4)
k. Worrying that doctors and nurses will not do what is right	(0)	(1)	(2)	(3)	(4)
l. Getting a thorough examination	(0)	(1)	(2)	(3)	(4)

m.	Feeling like doctors are trying to give as little service as possible	(0)	(1)	(2)	(3)	(4)
n.	Uncaring office staff	(0)	(1)	(2)	(3)	(4)
o.	Getting your questions answered	(0)	(1)	(2)	(3)	(4)
p.	Not knowing what to expect from one visit to the next	(0)	(1)	(2)	(3)	(4)
q.	Doctors rushing you through the visit	(0)	(1)	(2)	(3)	(4)
r.	I am not confident the mobile clinic will be in my neighborhood long-term	(0)	(1)	(2)	(3)	(4)
s.	Lack of privacy	(0)	(1)	(2)	(3)	(4)
t.	I don't think the mobile clinic has everything needed to provide complete care.	(0)	(1)	(2)	(3)	(4)

18. Do you use a COMMUNITY CLINIC (for example: Daughters of Charity, St. Thomas, EXCELth, Jefferson Community Clinics, St. Charles Community Health Center)?

1☐ Yes

2☐ No

Please answer #19, regardless of your answer to #18.

19. People often face barriers when trying to get health care. Do you think these would be problems going to a <u>COMMUNITY CLINIC</u>?

Problems with:		Big problem	Somewhat of a problem	Unsure	Not a big problem	Not a problem at all
a.	Getting to the community clinic	(0)	(1)	(2)	(3)	(4)
b.	Getting hold of the community clinic by phone	(0)	(1)	(2)	(3)	(4)
c.	Having to wait too many days to be seen	(0)	(1)	(2)	(3)	(4)
d.	Getting care after hours or on the weekends	(0)	(1)	(2)	(3)	(4)
e.	Having to take care of household responsibilities	(0)	(1)	(2)	(3)	(4)
f.	Having to take time off work	(0)	(1)	(2)	(3)	(4)
g.	Doctors or nurses not fluent in your language	(0)	(1)	(2)	(3)	(4)
h.	Getting referrals to specialists	(0)	(1)	(2)	(3)	(4)
i.	Getting enough help with paperwork or forms	(0)	(1)	(2)	(3)	(4)
j.	Mistakes made by doctors or nurses	(0)	(1)	(2)	(3)	(4)

k. Worrying that doctors and nurses will not do what is right	(0)	(1)	(2)	(3)	(4)
l. Getting a thorough examination	(0)	(1)	(2)	(3)	(4)
m. Feeling like doctors are trying to give as little service as possible	(0)	(1)	(2)	(3)	(4)
n. Uncaring office staff	(0)	(1)	(2)	(3)	(4)
o. Getting your questions answered	(0)	(1)	(2)	(3)	(4)
p. Not knowing what to expect from one visit to the next	(0)	(1)	(2)	(3)	(4)
q. Doctors rushing you through the visit	(0)	(1)	(2)	(3)	(4)
r. I am not confident the community clinic will be in my neighborhood long-term	(0)	(1)	(2)	(3)	(4)
s. Lack of privacy	(0)	(1)	(2)	(3)	(4)
t. I don’t think the community clinic has everything needed to provide complete care.	(0)	(1)	(2)	(3)	(4)
